# Analysis of Delayed Vaccination Regimens: A Mathematical Modeling Approach

**DOI:** 10.3390/epidemiologia2030021

**Published:** 2021-07-20

**Authors:** Gilberto Gonzalez-Parra

**Affiliations:** Department of Mathematics, New Mexico Tech, New Mexico Institute of Mining and Technology, Socorro, NM 87801, USA

**Keywords:** SARS-CoV-2 virus, vaccination, delayed dose, mathematical modeling, simulation

## Abstract

The first round of vaccination against coronavirus disease 2019 (COVID-19) began in early December of 2020 in a few countries. There are several vaccines, and each has a different efficacy and mechanism of action. Several countries, for example, the United Kingdom and the USA, have been able to develop consistent vaccination programs where a great percentage of the population has been vaccinated (May 2021). However, in other countries, a low percentage of the population has been vaccinated due to constraints related to vaccine supply and distribution capacity. Countries such as the USA and the UK have implemented different vaccination strategies, and some scholars have been debating the optimal strategy for vaccine campaigns. This problem is complex due to the great number of variables that affect the relevant outcomes. In this article, we study the impact of different vaccination regimens on main health outcomes such as deaths, hospitalizations, and the number of infected. We develop a mathematical model of COVID-19 transmission to focus on this important health policy issue. Thus, we are able to identify the optimal strategy regarding vaccination campaigns. We find that for vaccines with high efficacy (>70%) after the first dose, the optimal strategy is to delay inoculation with the second dose. On the other hand, for a low first dose vaccine efficacy, it is better to use the standard vaccination regimen of 4 weeks between doses. Thus, under the delayed second dose option, a campaign focus on generating a certain immunity in as great a number of people as fast as possible is preferable to having an almost perfect immunity in fewer people first. Therefore, based on these results, we suggest that the UK implemented a better vaccination campaign than that in the USA with regard to time between doses. The results presented here provide scientific guidelines for other countries where vaccination campaigns are just starting, or the percentage of vaccinated people is small.

## 1. Introduction

The spread of SARS-CoV-2 has reached almost every region of the world and has caused a global health issue with more than 170 million confirmed cases and currently about 3.45 million deaths [1,2]. Many non-pharmaceutical health interventions have been deployed in many countries worldwide [3-6]. The complex process of SARS-CoV-2 spread is affected by several factors that are currently not very well understood [7-11]. Among important factors, we can include social behavior, age, weather variables, virus variants, and immunocompetence [12-14]. Thus, the study of the dynamics of the spread of SARS-CoV-2 is important to reduce the burden caused by the COVID-19 pandemic.

Currently, several countries such as the USA, the UK, and European countries have implemented different vaccination campaigns [15]. There are many different ways to design a vaccination program, taking into account temporal factors, vaccination rate, age, geographical factors, immune system, health status, and even type of vaccine [6,16-18]. Health institutions in the USA and the UK have implemented different vaccination strategies, and some researchers have been investigating the best options for vaccine campaigns [19]. Previous studies have found that the vaccination pace and the efficacy of the vaccine are the main factors in reducing the number of deaths, number of infected, and hospitalization [17,20,21]. Besides these aforementioned factors, a few researchers have studied the effect of delayed second doses on important health outcomes such as death, among others [6,19,22,23]. Forecasting the long-term dynamics of SARS-CoV-2 in a population is an extremely complex problem due to the great number of variables involved, including the non-pharmaceutical interventions affecting social contacts [24-27]. Forecasting the COVID-19 pandemic for a long period, including factors such as planned vaccine rollout and the appearance of new SARS-CoV-2 variants with different transmissibilities, is very challenging, as time has shown [20,28-34]. For other epidemics due to viruses such as influenza and respiratory syncytial virus, forecasting is less complex, since, even though social contacts are a factor, social behavior has not changed as much as it has during the COVID-19 pandemic [35-51]. Mathematical models allow us to study the effects of non-pharmaceutical interventions on important outcomes without the need to make forecasts. Thus, in this study, we are interested in studying different vaccination campaigns where times between doses are varied. In [23], the authors developed an agent-based model to investigate whether to vaccinate more individuals with the first dose of available vaccines and delay the second dose or to continue with the recommended two-dose series as tested in clinical trials. The authors found that for Moderna vaccines, a delay of at least 9 weeks could maximize the vaccination program’s effectiveness. In [6], the authors concluded that vaccination alone is insufficient to contain the outbreak. In another study, it has been recommended, based on population health outcomes, that a vaccination campaign with a delayed second dose versus the standard schedule of SARS-CoV-2 mRNA vaccination could result in reduced cumulative mortality under certain conditions [22]. In that interesting article, the authors used in their study 90%, 80%, and 70% first dose efficacy. They found that a delayed second dose strategy was optimal for vaccine efficacies at or above 80% and vaccination rates at or below 0.3% of the population per day.

In this study, we developed a mathematical model of COVID-19 transmission to compare the impact of different vaccination regimens on main health outcomes such as deaths, hospitalizations, and the number of infected. Our aim here is not to precisely forecast the COVID-19 pandemic but rather to understand the effects of using different vaccination strategies on key outcomes such as the number of infections, hospitalizations, or deaths. That is, we do not expect to forecast the number of infected people at the end of a given summer or the number of deaths in the USA. As we have mentioned, the long-term forecasting of the COVID-19 pandemic is very complex, as time has proven [29,31,32]. One main aim of this study is to gain insight into the optimal strategies regarding the number of weeks of delay for the second dose of the SARS-CoV-2 vaccine. As we have mentioned previously, there are other factors that can affect finding the optimal strategy, but here we focus on the time interval between doses and vaccination rate, which are variables that governments can relatively easily manage or design. A number of vaccines, including those developed by Pfizer–BioNTech, Moderna, and Oxford–AstraZeneca, have different efficacies after the first and second doses. Thus, we consider a variety of efficacies for these doses. Regarding the vaccination pace, this could be affected mainly by three factors. First, the availability of the vaccine. Second, the resources or health care workers related to the inoculation process. The third is related to the willingness of people to be vaccinated [20,52,53]. It is important to remark that clinical trials and evaluations of mass vaccination campaigns have demonstrated that these vaccines can provide high levels of protection against symptomatic and severe disease with two doses administered 3–4 weeks apart [23]. Thus, delaying a second dose could be riskier since those particular time delays between doses were not tested. However, the UK has implemented a 12-week delay for the second dose, and, based on the current results, it seems safe. Furthermore, the UK is no longer alone in its decision, with Canada and Germany both choosing to follow a similar plan [54]. Therefore, in this work, we consider a variety of time delays and study faster and broader population-level protection against COVID-19. In addition, it is important to mention that many countries currently have a low percentage of their population vaccinated, and obtaining scientific support to select the optimal vaccination campaign is of paramount importance.

A variety of mathematical models, statistical analyses, and computational techniques have been used extensively to study epidemic processes in the past and in particular now for the current COVID-19 pandemic [4,11,33,40,55-65]. One main advantage of mathematical models is that many different simulations can be completed, allowing the investigation of different factors under a variety of scenarios. In this article, we construct a compartmental mathematical model based on ordinary differential equations and use computational methodologies to study different scenarios. In the model developed here, we include the presymptomatic and asymptomatic carriers of SARS-CoV-2, who are nevertheless able to spread the virus. It has been mentioned that asymptomatic people are in some way a key contributor to the spread of the SARS-CoV-2 virus and are an important consideration for control policies [11,66-80]. In addition, we study scenarios with different SARS-CoV-2 virus transmission rates since the social situation is different depending on the region. This will generate different effective reproduction numbers *R_t_* of COVID-19 [81-84].

## 2. Materials and Methods

### Mathematical Model

We constructed a mathematical model that includes ordinary differential equations and takes into account vaccination processes. The model divides the total population into different classes depending on the COVID-19 progression and vaccination status. Thus, the considered classes or subpopulations are: susceptible, latently infected (not yet infectious), presymptomatic (and infectious), infected (able to infect others), asymptomatic (able to infect others), hospitalized, recovered (not infectious), vaccinated with one dose, and, lastly, vaccinated with two doses. The mathematical model constructed considers transitions of individuals through the aforementioned classes. We assume that recovered individuals have long immunity against reinfection during the period of study that is shorter than 1 year. We also assume that only susceptible individuals and those vaccinated with one dose are the only ones that can be vaccinated. In some way, this implies that testing rate is high, and since the size of the latent, presymptomatic, and asymptomatic subpopulations are small, qualitative outcomes from the study would not change. This study is not applicable for regions that are planning to use just one-dose vaccines.

The individuals can transit from the susceptible class to vaccinated (one dose) if they receive the vaccine. In an analogous way, individuals vaccinated with one dose can transit to the fully vaccinated (two doses). In this last case, we consider that the immunity is boosted, and the fully vaccinated individual has greater protection against SARS-CoV-2 in comparison with those with one dose [20,22,23,85]. The mathematical model, as with many others in epidemics, implicitly assumes that transitions follow an exponential distribution. Other, more realistic models, such as the Erlang distributions, are more complex with larger numbers of parameters [86-90]. However, in some cases, exponential distributions are not far from reality. We have found that the length of stay in hospital is not far from an exponential distribution [91]. The constructed model considers that only hospitalized individuals can die due to COVID-19 [4,91-93]. This last indicator is of paramount importance for the comparison of different vaccination strategies and is used in this study extensively [4,94-96].

We use a mathematical model that is similar to a classical SEIAR-type epidemiological model to explain the dynamics of SARS-CoV-2 spread under a vaccination program. The model has parameters related to the vaccine efficacy after one and two doses, vaccination pace, and interval time between doses. Thus, the values of these parameters can be varied in order to study many different scenarios. Being able to vary the parameter values is relevant since the efficacy of vaccines varies, and vaccination rates are different depending on the country or region [52,53,97-101].

The constructed mathematical model is given by

**Figure 1.**
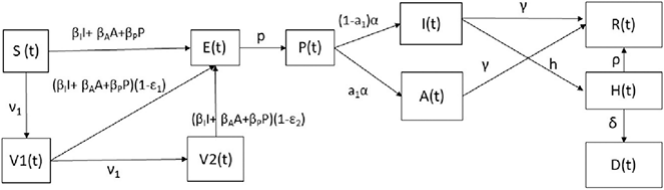
Diagram for the COVID-19 mathematical model (1). The boxes represent the different classes and the arrows the transitions between these classes.

(1) S.(t)=−(βII(t)+βAA(t)+βPP(t))S(t)N(t)−v1,V.1(t)={v1−(βII(t)+βAA(t)+βPP(t))(1−ϵ1)V1(t)N(t),(period1)−(βII(t)+βAA(t)+βPP(t))(1−ϵ1)V1(t)N(t)−v1,(period2)}V.2(t)={−(βII(t)+βAA(t)+βPP(t))(1−ϵ2)V2(t)N(t),(period1)v1−(βII(t)+βAA(t)+βPP(t))(1−ϵ2)V2(t)N(t),(period2)}E.(t)=(βII(t)+βAA(t)+βPP(t))(S(t)N(t)+(1−ϵ1)V1(t)N(t)+(1−ϵ2)V2(t)N(t))−pE(t)P.(t)=pE(t)−(α+d)P(t)A.(t)=aαP(t)−γA(t)I.(t)=(1−a)αP(t)−γI(t)H.(t)=hI(t)−(δ+ρ)H(t)R.(t)=γ(I(t)+A(t))+ρH(t)D.(t)=δH(t)

where *S*(*t*) represents the susceptible class. When a susceptible and an infectious individual comes into infectious contact, the susceptible individual contracts the disease and transitions to the latent class *E*(*t*). Individuals in class *E*(*t*) are infected but not yet infectious. The presymptomatic (infectious) class *P*(*t*) includes infectious individuals without symptoms, and they transit later on to the asymptomatic and infected subpopulations. The classes *I*(*t*) and *A*(*t*) include infected (able to infect others) and asymptomatic carriers (able to infect others), respectively. The subpopulation *H*(*t*) includes the hospitalized individuals at time *t*. The compartment *D*(*t*) represents the number of deaths due to the SARS-CoV-2 virus from the beginning of the simulation period. The model assumes that people in states *E*(*t*), *H*(*t*), and *R*(*t*) are not able to transmit SARS-CoV-2. In addition, we consider two subpopulations of vaccinated people: the first *V*_1_(*t*) includes the individuals that have been given one dose, and *V*_2_(*t*) includes those fully vaccinated with two doses. Based on the efficacies of the vaccines and doses, those vaccinated can become infected. The model assumes a constant vaccination rate per day, i.e., the same number of doses per day. Some countries have specific goals for the number of inoculations per day, but this is difficult to achieve in practice and requires a great amount of resources and vaccine availability. To attain a constant vaccination rate in the simulation involves some complexity from a mathematical and computational point of view since special care needs to be given to avoid negative values for the subpopulations. We deal with this by setting a condition in the model so that all subpopulations will have non-negative values. This particular design can be interpreted in that if there is no population in *V*_1_(*t*) (one dose), then the model will not permit the vaccination of more individuals from *V*_1_(*t*). For the subpopulation *S*(*t*), the condition is not as pertinent, since there is a great number of susceptible persons. Therefore, the simulation stops before the susceptible subpopulation *S*(*t*) is depleted due to the fact that some individuals are reluctant to be vaccinated. One remarkable characteristic of the proposed mathematical model is the use of different expressions for the derivatives of the variables representing the two subpopulations of vaccinated people *V*_1_(*t*) and *V*_2_(*t*). This implies that there is a perfect synchronization of inoculations between the first and the second doses; the model will use the first expression for *V*_1_(*t*) and the first expression of *V*_2_(*t*) at the same time period, i.e., vaccination of only susceptible people in time period 1. Then, the numerical simulation uses the second expression for *V*_1_(*t*) and the second expression of *V*_2_(*t*) at the second time period; i.e., vaccination of only partially vaccinated people in time period 2. This process alternates until the end of the time period of study. Thus, time periods 1 and 2 would change depending on the vaccination regimen that has been deployed. The diagram of the mathematical model without this particular approach is presented in Figure 1.

The parameter values used for this study are taken from the scientific literature, even though there are some discrepancies with regard to some values. In this study, we are mainly interested in the impact of the temporal regimen of vaccination. However, another factor that needs to be taken into account is the vaccination pace, which, in turn, is dependent on the vaccine’s availability and the supply process. In addition, the efficacy of the vaccine as a function of the brand is also a variable that affects the outcomes regarding infected, hospitalized, and death cases. During this study, we assume that the transmission rates of SARS-CoV-2 are constant from the beginning of the period of study. Implicitly, this assumes that social behavior is in a steady state after a long time period. However, it has been mentioned that people might change behavior (on average) when a vaccination program in a country is well advanced due to a decreasing feeling of risk [6]. Nevertheless, we assume that social contacts would not change because many health policies and guidelines would have already been implemented long before the vaccination program had begun. Thus, as in other studies, we take an approximation and a conservative assumption that the transmissibility would not change during the deployment of the vaccination program. In addition, some studies have indicated that the antibody titers may decline over time in patients recovered from COVID-19, particularly in those who were asymptomatic [102]. However, we do not consider that recovered individuals can return to the susceptible stage. One reason for this is that the time horizon of this study is less than 1 year, and, based on preliminary results, it seems plausible that immunity lasts more than 6 months [103]. We also consider that for this time interval, the immunity provided by the vaccines does not diminish.

Regarding the scenarios considered, we take into account the situation in the USA before the vaccination program started. However, since we are interested in qualitative results instead of forecasts, the results presented here can be extrapolated to other countries. Thus, we considered vaccination rates from 100,000 to 2 million inoculations per day in this study. The vaccination rate in the USA has been variable, and forecasting this rate beforehand in any country would seem to be overconfident. For instance, US federal officials hoped for 20 million people to receive their first of two required shots by the end of 2020. However, that goal was changed, and just over 1 million doses of vaccines were administered by the first of January 2021 [104]. Another aspect that needs to be taken into account in the real world is the reluctance of some people to be vaccinated, and this could disturb any vaccination rate or plan [53]. For the death rate of hospitalized individuals, we use a variety of data from the scientific literature [4,17,105-107]. However, as we mentioned before, the qualitative results would not change if different death rates are assumed. For the asymptomatic cases and proportions, we used data from the scientific literature which have discrepancies [4,69,73,108-112]. We chose a conservative approach wherein which the percentage of infections that are asymptomatic infections is 50% [1,113]. In addition, we included an extreme case (from CDC scenarios) in which this percentage is only 15%, which the CDC defines as not the best estimate [113]. However, as we mentioned before, the qualitative results would not change even for this extreme case. Similarly, these qualitative results do not change if different reasonable values are assumed for parameters other than key ones such as vaccine efficacy, vaccination rate, and transmission rates. For the parameters *β_P_*, *β_I_*, and *β_A_*, we also assume a conservative approach with similar values as has been used in some studies [4,73,108,109,112,114].

For the initial conditions, we assume the particular situation of the USA since data here, in general, is more reliable. Transforming the data to proportions or to a standard 10,000 population, as in other studies, would not change the qualitative results. Thus, we set the initial conditions to the ones presented in Table 1. All the initial vaccinated subpopulations are set to zero since the simulations are performed at the beginning of the vaccination program, although this may not be true for a country in which a consistent and strong vaccination program is suddenly begun. More details about how to set the initial conditions can be found in [20]. For the initial susceptible subpopulation, we use a simple fact that initially there are no vaccinated individuals and therefore

(2) S(0)=N(0)−E(0)−P(0)−I(0)−A(0)−R(0)−H(0).

In Table 1, we present the initial conditions for the subpopulations.

## 3. Results

We present numerical results of the simulations of different scenarios. We vary the estimates of single- and two-dose efficacies on the main outcomes. In addition, we vary the rates of vaccination, which are related to vaccine availability and inoculation logistics. We consider three different vaccine regimens in which the time interval between the first and the second doses is varied (4, 8, and 12 weeks). A time of 4 weeks has been called a standard regimen since most of the clinical trials were performed using this interval [15,19,22,23]. The other intervals were chosen based on the fact that the UK has adopted a maximal 12-week delay [15,54]. Regarding the transmission rate of SARS-CoV-2, it is well known that this varies depending on non-pharmaceutical interventions and physical contact. We considered several cases, but we present two main cases where the transmission rate is such that one case provides a basic reproduction number below one and another above one. Relatively small modifications of the particular values for the transmission rates do not change the qualitative results. The numerical simulations are based on the (1) mathematical model and allow us to analyze the main outcomes under different scenarios. We use the parameter values of Table 2 and the initial conditions given in Table 1. The numerical simulations and plots were processed using the Python programming language, in particular the scipy.odeint() function, which is a Python wrapper for the ODEPACK solver. It was not necessary to design a numerical scheme to guarantee the positivity of the variables. In other cases, it is necessary to construct numerical schemes to obtain realistic positive values [115-117].

The clinical trials of the Pfizer–BioNTech and Moderna vaccines involved two injections given 3–4 weeks apart, and both vaccines had approximately 95% efficacy after the second dose [19]. Nevertheless, by the day of the injection of the second dose, the efficacy of the first dose was somewhere in the range of 80–90% [19]. A vaccine efficacy of 87% from a single dose has been mentioned in previous work [22]. The CDC estimated a single-dose efficacy of either BNT162b2 or mRNA-1273 to be 80% [22,122]. However, there are other results that show different efficacies [97,123-130]. Moreover, other vaccines show different efficacies after the first and second doses. As well, the efficacies of the vaccines vary depending on the prevalent SARS-CoV-2 variant [131-134]. Thus, we consider a variety of scenarios with different efficacies for the vaccines. We include some cases where the efficacy of the vaccine could be low in order to consider the possibility that the available vaccine in one country might have low efficacy against one particular SARS-CoV-2 variant.

We also vary the inoculation rate (vaccination pace) to test different potential vaccination program scenarios. It is important to remark that despite the plans that health institutions make regarding vaccination, there are uncertainties present in the inoculation process [135,136]. Taking into account the situation of the USA, at the beginning of the vaccination program, we consider inoculation rates of 100,000 and 2 million per day. It is important to mention that even though these rates vary, this study gives insights into the impact of vaccine regimens on critical outcomes of the COVID-19 pandemic.

**Figure 2.**
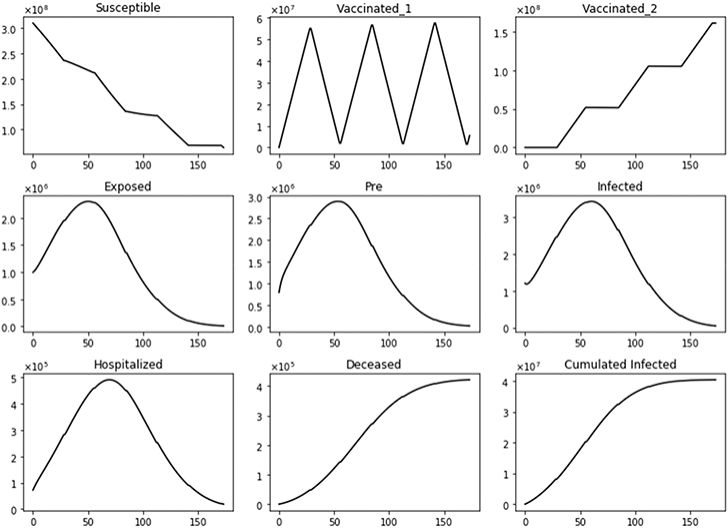
Dynamics of several subpopulations under a standard vaccine regimen of 4 weeks between doses. This scenario considers a transmission rate such that the basic reproduction number *R*_0_ is greater than one, the vaccination pace is 2 million per day, and the vaccine efficacy after the first dose is 60% and 95% after the second dose.

Figure 2 shows the dynamics of several subpopulations under a standard vaccine regimen of 4 weeks between doses. It can be observed that the susceptible subpopulation decreases both because some of this population acquire SARS-CoV-2 and others are inoculated with the first dose. Thus, the susceptible subpopulation always decreases. This particular scenario considers a transmission rate that gives a basic reproduction number *R*_0_ greater than one, a vaccination pace of 2 million per day, the vaccine efficacy after the first dose of 60%, and 95% after the second. Under these conditions, the infected people increase initially and then decrease due to the protective effect of the vaccine. The population vaccinated with a single dose increases in certain time periods, but during others decreases, since after 4 weeks, the population vaccinated with one dose receives the second and thus transits to the fully vaccinated subpopulation *V*_2_(*t*). On the other hand, the number of fully vaccinated people always increases or stays constant. This last aspect is due to the fact that the model does not consider a waning effect for a period of less than 1 year, plus the fact that the model has no demographics included due to the short time period of study. For a short time period, not all individuals will pass through compartment E. In fact, the majority of subjects will end up in the subpopulation *V*_2_ (vaccinated with two doses), and in the recovered, R. The exact distribution at the end of the simulation depends on the transmission rates and the efficacies of the vaccines. Transitions from compartments *V*_1_ and *V*_2_ to *E* are due to the inefficacy of the vaccines (some vaccinated can still be infected).

**Figure 3.**
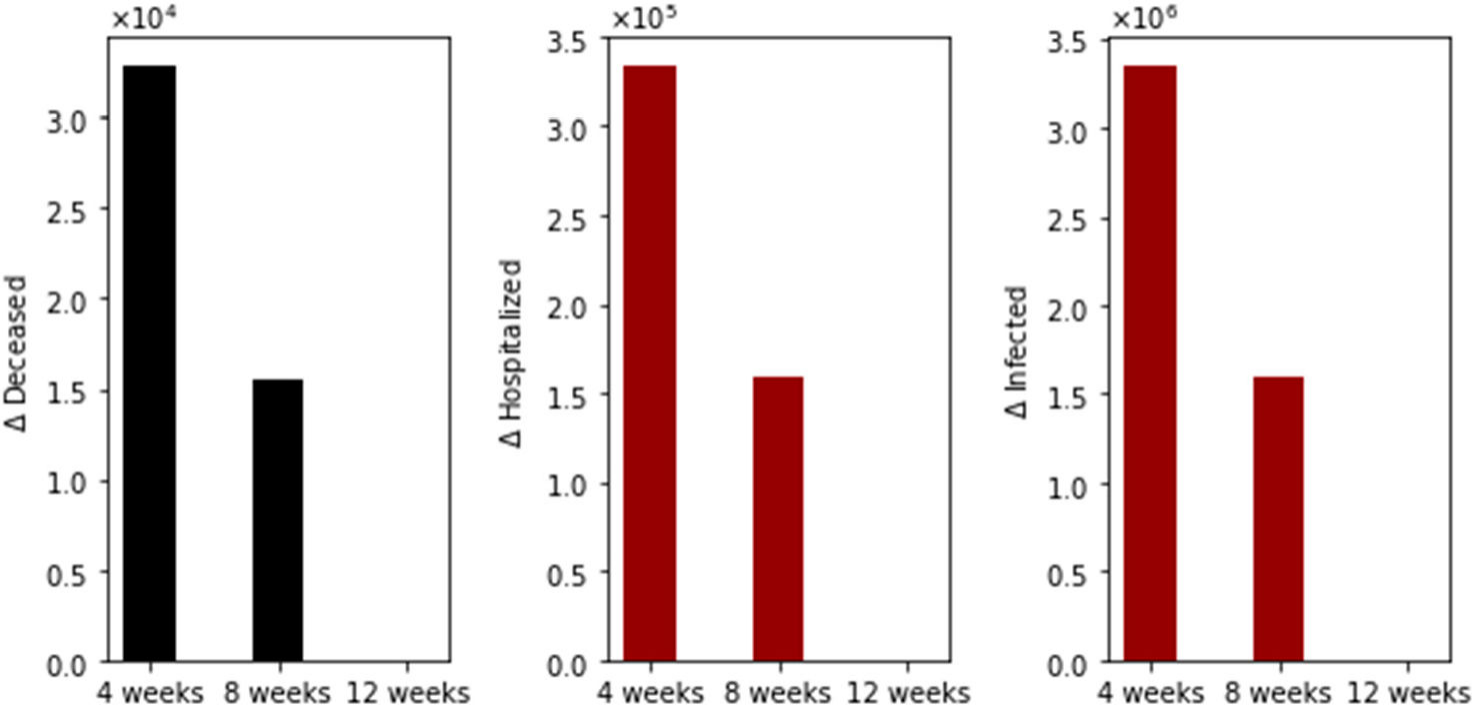
Differences in the total number of deaths, hospitalized, and infected, under different time interval vaccine regimens. This scenario considers a transmission rate such that the basic reproduction number *R*_0_ is greater than one, the vaccination pace is 2 million per day, and the vaccine efficacy after the first dose is 60% and 95% after the second.

With regard to main outcomes, Figure 3 shows the difference in the total number of deaths, hospitalized, and infected, under the different time interval vaccine regimens. Under this particular scenario, there are more total deaths for the standard (4-week interval) vaccination regimen in comparison with the 8- plus 12-week regimens. Moreover, the total deaths of the 8-week vaccination regimen are greater than that of the 12-week regimen. The same situation happens with the total number of hospitalized and infected individuals, as expected. This initial result agrees partially with two interesting recent publications where the authors used agent-based stochastic models [21-23].

**Figure 4.**
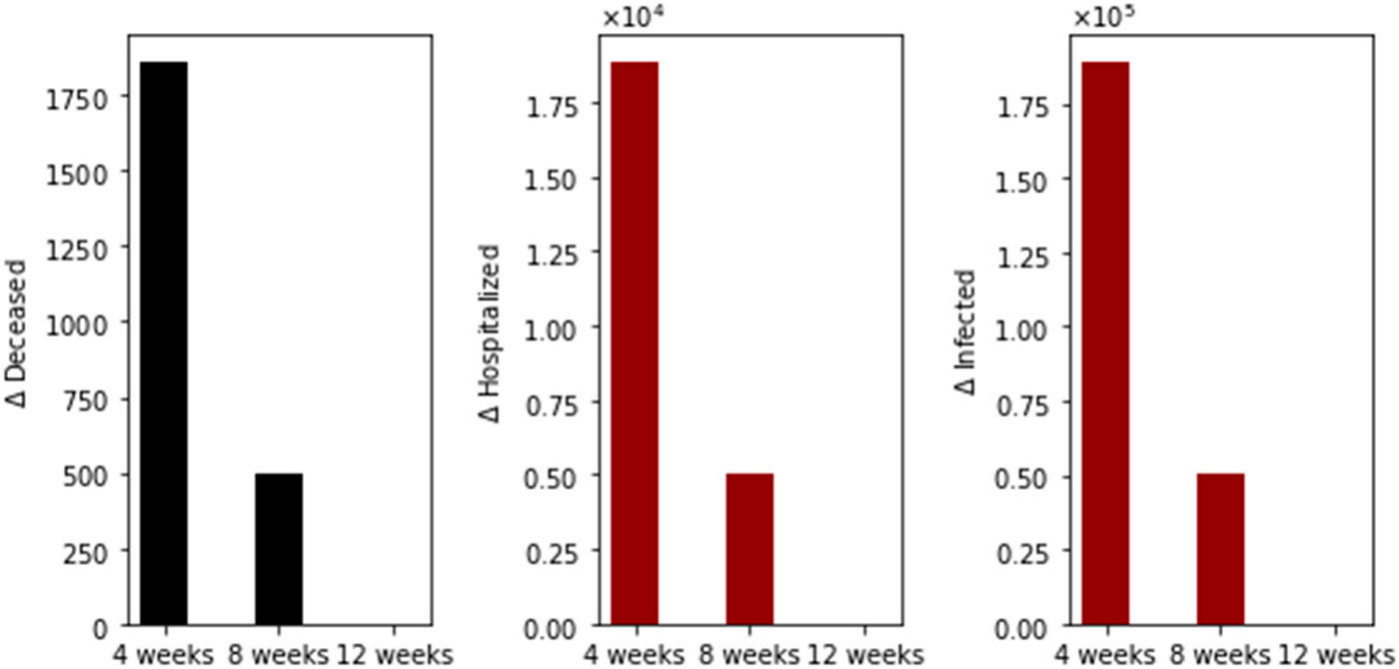
Differences in the total number of deaths, hospitalized and infected, under the different time interval vaccine regimens. This scenario considers a transmission rate such that the basic reproduction number *R*_0_ is less than one, the vaccination pace is 2 million per day, and the vaccine efficacy after the first dose is 60% and 95% after the second dose.

We performed additional numerical simulations varying the transmission rate in order to consider scenarios where the basic reproduction number *R*_0_ is less than one. The other conditions are the same. The vaccination pace is 2 million per day; the vaccine efficacy after the first dose is 60% and 95% after the second. In Figure 4, we show the differences in the total number of deaths, hospitalized, and infected, under the different time interval vaccine regimens. Again, the standard vaccination regimen produces a total number of deaths greater than the other vaccination regimens, and the 8-week vaccination program produces more total deaths than the 12-week program. In this case, we have fewer deaths for all the vaccination regimens, as was expected since the transmissibility is lower.

**Figure 5.**
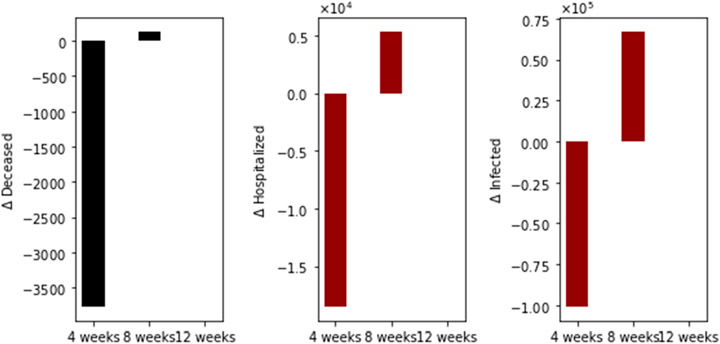
Differences in the total number of deaths, hospitalized, and infected, under the different time interval vaccine regimens. This scenario considers a transmission rate such that the basic reproduction number *R*_0_ is greater than one, the vaccination pace is just 100,000 per day, and the vaccine efficacy after the first dose is 60% and 95% after the second dose.

**Figure 6.**
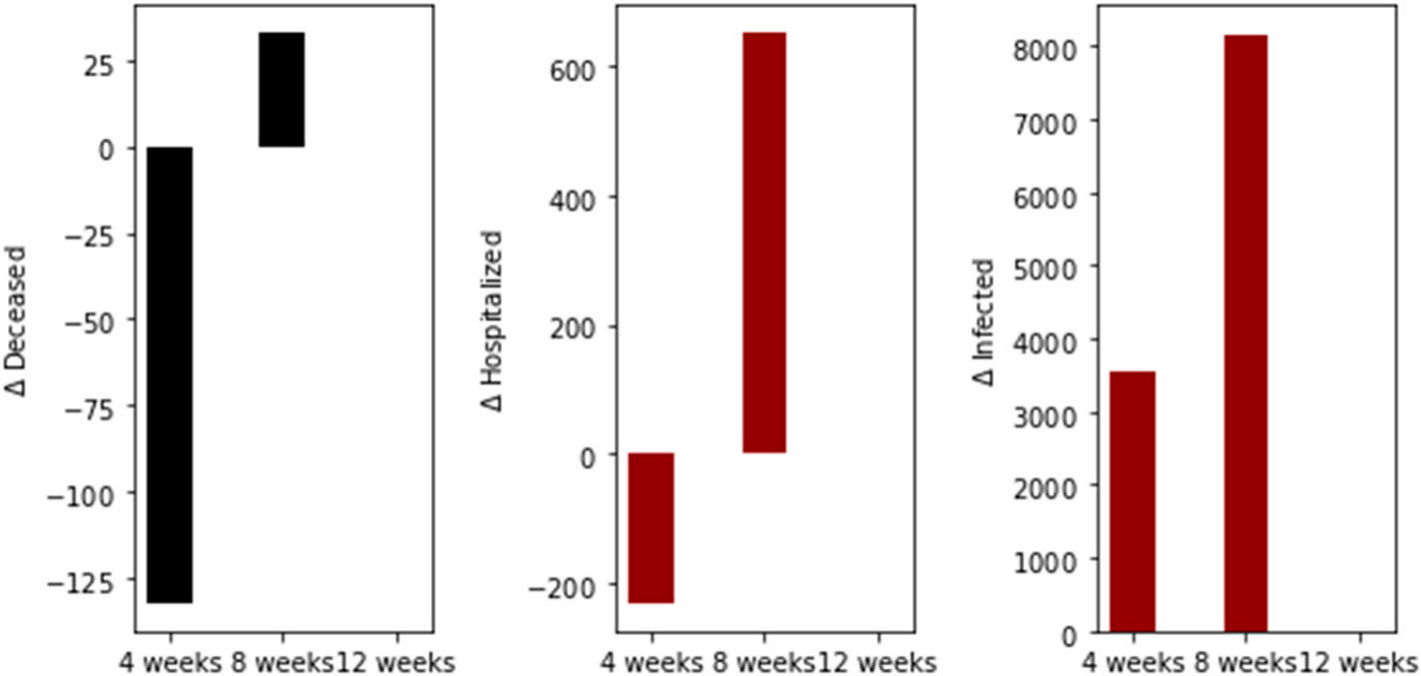
Differences in the total number of deaths, hospitalized, and infected, under the different time interval vaccine regimens. This scenario considers a transmission rate such that the basic reproduction number *R*_0_ is less than one, the vaccination pace is just 100,000 per day, and the vaccine efficacy after the first dose is 60% and 95% after the second dose.

**Figure 7.**
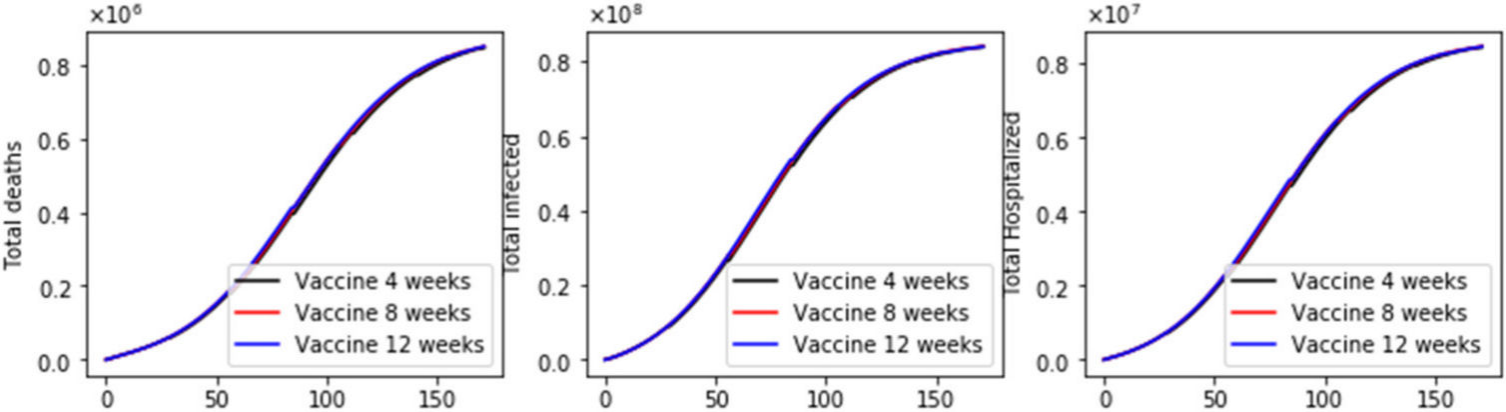
Dynamics of the total number of deaths, hospitalized, and infected, under the different time interval vaccine regimens. This scenario considers a transmission rate such that the basic reproduction number *R*_0_ is greater than one, the vaccination pace is just 100,000 per day, and the vaccine efficacy after the first dose is 60% and 95% after the second dose.

We performed two additional simulations varying the vaccination rate (*ν*_1_ = 100,000) since this is also a factor that affects a vaccination program [17,20]. Thus, in Figures 5 and 6, we present two results with a basic reproduction number *R*_0_ greater and less than one, respectively. In both cases, the standard vaccination regimen produces fewer total deaths. However, the differences are smaller in comparison when 2 million doses per day were administered, as can also be seen in Figure 7. These last results are interesting since, in some way, they support an opposing view from the previous. Thus, for low vaccination rates, the cycles of the vaccination regimens are more crucial, which could explain the differences when the vaccination pace is much higher. We will perform additional simulations with a variety of plausible scenarios in order to see if there are patterns that allow us to reach a stronger conclusion about the vaccination regimens.

**Figure 8.**
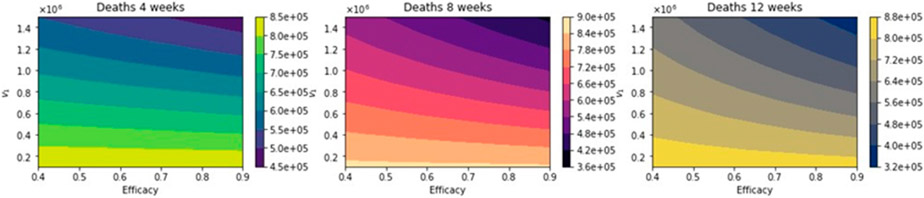
Total number of deaths for a variety of vaccine efficacies after the first dose and different vaccination rates per day. The basic reproduction number *R*_0_ > 1.

**Figure 9.**
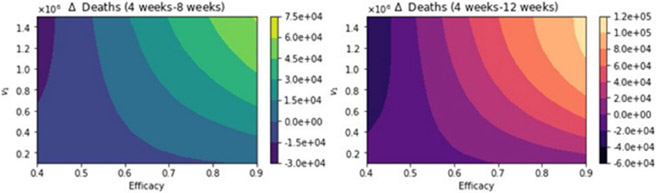
Difference between the total number of deaths for the different vaccination regimens, and under a variety of vaccine efficacies after the first dose and different vaccination rates per day. The basic reproduction number *R*_0_ > 1.

Figure 8 shows the total number of deaths for a variety of vaccine efficacies after the first dose and different vaccination rates per day. As expected, the total number of deaths decreases significantly with an increase in the vaccination rate from 100,000 inoculations per day to 1.5 million. In addition, it can be seen that the number of deaths also decreases for higher vaccine efficacy. However, our main interest is finding which of the three vaccination regimens gives the least total number of deaths, hospitalizations, and infected. Thus, in Figure 9, we show the difference between the vaccination regimens regarding the total number of deaths. It can be seen that for lower vaccine efficacies of the first dose, the standard regimen is better than the other vaccine regimens. The explanation for this is that if a first dose of the vaccine is low, it is better to inoculate the people with a second dose as fast as possible due to a high efficacy of 95% of the second dose. With regard to the vaccination rate, it can be observed that a higher vaccination rate under a high first-dose efficacy favors the 12-week vaccination regimen. This is due to the fact that one dose would produce reasonable protection against SARS-CoV-2, and the more people vaccinated with a first dose, the larger the difference in deaths with respect to the standard vaccination regimen. Thus, these results agree with previous results obtained despite the variation in some assumptions and models [21-23]. However, for a fixed efficacy of the first dose of 60%, an increase of the vaccination rate would shift the optimal vaccination regimen from the standard one (4 weeks) to the delayed one of 12 weeks. This behavior is not easy to grasp without a mathematical modeling approach and is due in some way to the nonlinearity of the spread of SARS-CoV-2. The nonlinearity is present in the contact between susceptible and infected individuals and has been correctly taken into account in previous studies regardless of the type of model [21-23].

**Figure 10.**
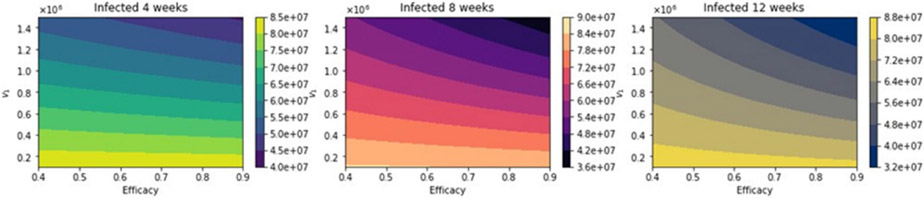
Total number of infected people for a variety of vaccine efficacies after the first dose and different vaccination rates per day. The basic reproduction number *R*_0_ > 1.

**Figure 11.**
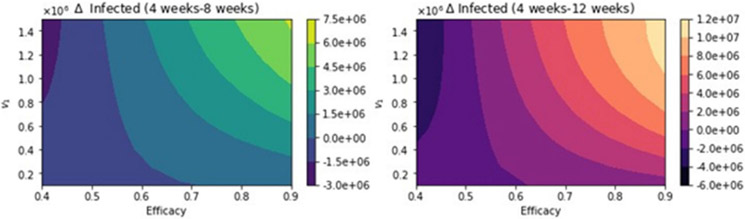
Differences between the total number of infected for the different vaccination regimens, and under a variety of vaccine efficacies after the first dose and different vaccination rates per day. The basic reproduction number *R*_0_ > 1.

With regard to total infected cases, the results are similar to those for total deaths. Figure 10 shows the total number of infected people. Again, as expected, the total number of infected decreases with an increase in the vaccination rate and decreases for higher vaccine efficacy. Figure 11 shows the difference between the vaccination regimens regarding the total number of infected people. Again, it can be seen that not for all cases, a 12-week vaccination regimen produces fewer infected people. However, for a first-dose vaccine efficacy of 60% and a vaccination rate of more than 300,000 per day, the 12-week vaccination regimen produces a lower number of infected. In a previous study where the authors used a stochastic agent-based model, they simulated scenarios with a first-dose vaccine efficacy in the range of 50—90%, and a vaccination rate in the range of 0.1 — 1% of population per day [22]. These ranges are close to those that we have used in the proposed scenarios studied. The authors in [22] found that a delayed second dose strategy was optimal for vaccine efficacies at or above 80% and vaccination rates at or below 0.3%. Regarding the vaccination rates, we obtained a different result since, in our simulations, a higher vaccination rate produces less cumulative mortality for the delayed 12-week vaccination regimen. However, our results agree well with those found in [21] in that the delayed-vaccination regimen is better for higher vaccination rates. We will discuss these results further in the next section.

Based on the previous results, we can conclude that under some plausible scenarios where the vaccine efficacy after the first dose is above 80%, and the vaccination rate is significant, the vaccination regimen of 12 weeks generates better outcomes than the standard 4-week vaccination regimen. The same conclusion has been reached when the vaccination regimen of 8 weeks is compared with one of 12 weeks. However, for vaccines with a low efficacy after the first dose, the standard vaccine regimen of 4 weeks would produce lower cumulative mortality. Moreover, if the efficacy after the first dose is very low, then increasing the vaccination rate would be a worse option than the delayed 12-week vaccination regimen. This is due to the fact that, in some way, this regimen is delaying the good protection of 95% of the second dose.

### Further Uncertainty Analysis

**Figure 12.**
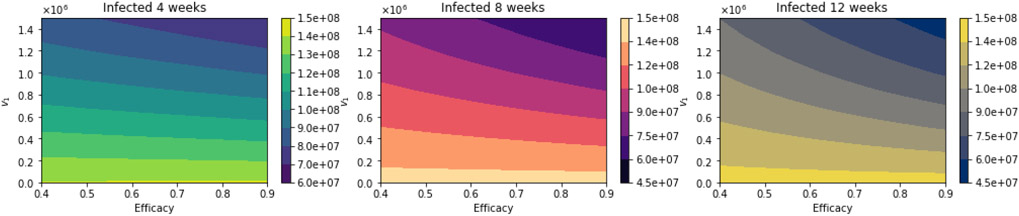
Total number of infected people for a variety of vaccine efficacies after the first dose and different vaccination rates per day. The basic reproduction number *R*_0_ > 1. Assuming that the percentage of infections that are asymptomatic is just 15% [113].

**Figure 13.**
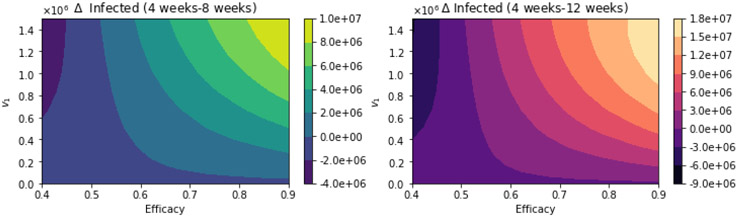
Differences between the total number of infected for the different vaccination regimens, and under a variety of vaccine efficacies after the first dose and different vaccination rates per day. The basic reproduction number *R*_0_ > 1. Assuming that the percentage of infections that are asymptomatic is just 15% [113].

We present here further uncertainty analysis not related to the vaccination program parameters. We include some additional analysis related to the parameters that might have more uncertainty, and we use CDC scenario guidelines that include some extreme cases [113]. The first case that we present is assuming that the percentage of infections that are asymptomatic is just 15% [113]. Figure 12 shows the total number of infected people for a variety of vaccine efficacies after the first dose and different vaccination rates per day. The total number of infected people decreases with an increase in the vaccination rate from 100,000 inoculations per day to 1.5 million. In addition, it can be seen that the number of infected people also decreases for higher vaccine efficacy. Figure 13 shows the main result regarding the delayed-dose strategy. For higher vaccine efficacies of the first dose and higher vaccination pace, the delayed 12-week vaccination regimen produces better outcomes under vaccination programs that have approximately efficacies above 60% and vaccination pace above 200,000 doses per day. From Figure 13, it can be seen that there is a threshold-level-curve that defines when one strategy is better versus another. Similarly, the same qualitative situation arises for deaths.

The second and last case is when we assume that the asymptomatic and presymptomatic individuals have less infectiousness relative to symptomatic ones. We again use the CDC as a guideline to chose the extremest case. We assume that the asymptomatic and presymptomatic individuals have just 25% of infectiousness relative to symptomatic people. It is important to remark that scientific literature shows a great uncertainty in this aspect, but the CDC considers that the best estimate is 75% [113].

**Figure 14.**
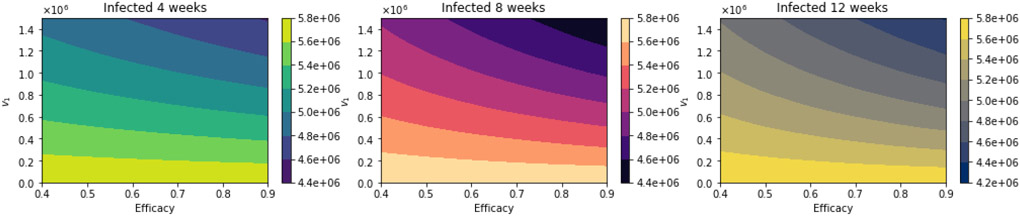
Total number of infected people for a variety of vaccine efficacies after the first dose and different vaccination rates per day. The basic reproduction number *R*_0_ > 1. Assuming that the asymptomatic and presymptomatic individuals have just 25% of infectiousness relative to symptomatic people [113].

**Figure 15.**
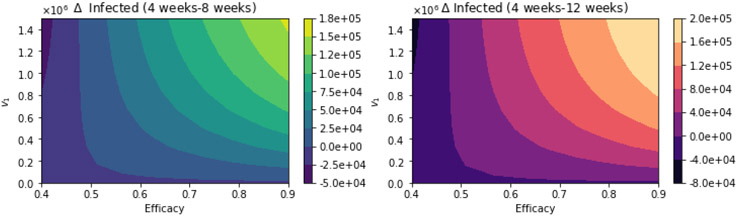
Differences between the total number of infected for the different vaccination regimens, and under a variety of vaccine efficacies after the first dose and different vaccination rates per day. The basic reproduction number *R*_0_ > 1. Assuming that the asymptomatic and presymptomatic individuals have just 25% of infectiousness relative to symptomatic people [113].

Figure 14 shows the total number of infected people for a variety of vaccine efficacies after the first dose and different vaccination rates per day. The total number of infected people decreases with an increase in the vaccination rate. In addition, it can be seen that the number of infected people also decreases for higher vaccine efficacy. Figure 15 shows the main result. For higher vaccine efficacies of the first dose and higher vaccination pace, the delayed 12-week vaccination regimen produces better outcomes under vaccination programs that have approximately efficacies above 60% and vaccination pace above 200,000 doses per day. Thus, under a great variety of scenarios, a delay on the second dose provides better outcomes. We performed additional simulations varying different parameters not related to the vaccination program parameters, such as the infectious period. Increasing the length of that gives similar qualitative results, and from a quantitative point of view, the outcomes are more remarked since infected people would infect more individuals. The results presented here are subject to the general limitations of the mathematical models. Further discussion is provided in the next two sections.

## 4. Discussion

There are several vaccines available around the world against the disease produced by SARS-CoV-2. Some countries have already started vaccination programs, including the USA, the UK, and Israel. Not all countries have deployed the same vaccination program and have used vaccines produced by different companies. Most of the vaccines require a second dose to achieve higher protection against the disease caused by SARS-CoV-2 [1,15,97,123,124,129,130]. Vaccination programs in some countries have not started yet, and in other countries, the number of vaccinated people is very low. It has been found that different vaccination programs produce different outcomes [17,20-23,52]. The design of an optimal vaccination program is a very complex problem due to the large number of variables involved and the nonlinearities included in the spread of SARS-CoV-2. For instance, a vaccination program might focus first on health care workers or on elderly people [85]. Thus, several health institutions from different countries have considered or implemented different vaccination regimens. Some of these regimens include prioritizing single-dose vaccination to reach a larger population faster. However, under a limited vaccine supply, this means delaying a second dose in comparison with a standard vaccination regimen of 4 weeks. This strategy has been controversial since most of the clinical trials were performed using a 4-week time interval between doses [15,19,22,23]. One remarkable world case is the UK, where the health authorities adopted a maximal 12-week delay [15]. The arguments in favor of a delayed second dose are on the basis of previous immunologic research that obtained significant protection against COVID-19 after just one dose [21,22].

In this study, we constructed a mathematical model to obtain insight into the complex problem of designing an optimal vaccination program based on vaccine efficacy, vaccine availability, and the time interval between the first and the second doses. We assessed the vaccination regimens using important health outcomes such as cumulative mortality, cumulative hospitalizations, and cumulative infected. The study of different COVID-19 vaccination programs is of paramount importance to reduce the burden of the COVID-19 pandemic [19,20,22,23,137-139].

For all the scenarios studied, we fixed the efficacy of the vaccines after the first dose to 95% since two of the most popular vaccines, such as Pfizer–BioNTech and Moderna have an efficacy of approximately 95% after the second dose [19]. This helps to focus on fewer factors. With regard to the efficacy of the vaccine after the first dose, we considered a variety of efficacies due to the high variability in the clinical trials and the appearance of SARS-CoV-2 variants [19,20,22,23,30,97,123-130,134,140-142]. In addition to the aforementioned factors, we included a variety of vaccination rates in order to consider different vaccine supplies in different countries. As we have mentioned before, there are many options for vaccination regimens. We focused on three: a standard vaccine regimen of 4 weeks between doses, another of 8 weeks, and a third of 12 weeks. The transmission rate under the current COVID-19 pandemic is time-dependent. Making a study with a time-dependent transmission rate is extremely complex due to the many options available and the high variability of social behavior of people under non-pharmaceutical interventions. However, with a mathematical approach, we can obtain useful insights into the optimal health policies regarding vaccination programs. Thus, we considered two transmission rate scenarios: one in which the basic reproduction number *R*_0_ is greater than one, and another in which it is less than one.

Using a mathematical epidemiological model based on differential equations, we found that for low vaccine efficacies of the first dose, the standard regimen is better than the other vaccine regimens. This conclusion makes sense since low initial protection would not avert many infected cases as the second dose can. Second, we also found that for higher efficacies (in general greater than 60%), after the first dose, a delayed vaccination regimen would avoid more deaths than the standard vaccination regimen of 4 weeks. This particular result agrees well with previous studies despite the differences in assumptions and modeling approach [21-23]. These results are important since they provide robustness and reliability to the strategy of delaying the second dose.

One surprisingly relevant difference that we found between our results and some previously reported is that we observed that larger vaccination rates favor the delayed second-dose vaccine regimen. However, in some previous studies, there were limitations on the benefits of the delayed vaccine regimen for large vaccination rates [22,23]. Despite this, in [21], the authors found results similar to ours regarding the vaccination rate. In particular, they found that for scenarios that would deliberately disadvantage a delayed-type strategy, the delayed option produced better overall outcomes. The variety of scenarios considered here regarding vaccine efficacy and rate have not been studied before to the best of our knowledge. Thus, the results provide a useful insight towards finding an optimal vaccination regimen.

The results presented here also indicate that, given a high vaccine efficacy for the first dose, the greater the number of vaccinations with the first dose, the larger is the difference in deaths with respect to the standard vaccination regimen. It is important to remark that for a fixed vaccine efficacy of 60%, an increase of the vaccination rate would shift the optimal vaccination regimen from the standard one to the delayed one of 12 weeks. This result may be of paramount importance if a particular country has a vaccine with a low first dose efficacy and large vaccine availability. This behavior transition is partially due to the nonlinearity of the spread of SARS-CoV-2, as has been correctly taken into account in previous studies [21-23].

The results presented here are not a quantitative forecast about the number of infected or vaccinated people over time. Accomplishing that aim over a time period of more than 1 month is a very challenging task due to the high variability of the social behavior of people under non-pharmaceutical interventions [4,29,31-33,62,143]. Moreover, including in the mathematical model, an accurate vaccination rate is an extremely difficult task due to changes in government policies, weather factors, approval of new vaccines, and resources available for vaccine distribution. Nevertheless, varying the vaccination rates in a large range provides useful information towards improving and designing better vaccination programs that help to reduce the burden of the COVID-19 pandemic.

It is important to remark that we did not consider the complication of the reluctance of a certain sector of the population to vaccinate [27,53,144,145]. The simulations decreased the susceptible population to a number above 10 million. However, it is quite possible that in the real world, that number would not be achieved due to the aforementioned reluctance. [27,53,144,145]. The results also show that the optimal vaccination program can be chosen regardless of the transmission rates. As expected, under a higher SARS-CoV-2 transmission rate, the delayed second dose program in comparison with the standard one can avert more deaths. Nevertheless, under this last scenario, either program would generate more deaths, infected, and hospitalized individuals due to the higher transmission rate.

Mathematical models have natural limitations due to different reasons, including the complexity of the real world. Thus, the results of using mathematical models are dependent on the assumptions and the underlying data. For this type of study, it is important to highlight the limitations and hypotheses since media, health authorities, and people can reach wrong conclusions under a variety of scenarios. Nonetheless, the findings are useful to scientifically support health policies [146-155]. There are several assumptions and limitations in this study. One important assumption that is common with previous studies is the use of time-invariant inoculation rates and transmission rates. We assumed that a steady state of the social behavior has been reached before the start of the vaccination program and is maintained during the simulation. The mathematical model includes the assumption that the different vaccination regimens can be planned with a perfect synchronization between the first and the second doses. The mathematical model does not include demographic factors due to the short time period of study [58,156].

In this study, we did not consider vaccination programs that target specific age groups or other subpopulations such as health care workers. Further studies that include these factors would require more parameters and, therefore, more uncertainty and details. In addition, our mathematical model does not consider the fact that immunity due to the vaccine or the infection wanes over time. Although, even if immunity wanes, similar results have been obtained [21]. In this analysis, vaccines of just one dose were not studied since there is obviously no strategy for the time between doses. The SARS-CoV-2 variants and their specific contagiousness were not included in the mathematical model. However, based on the fact that it has been found that vaccines also give protection against new SARS-CoV-2 variants, we expect that our results can be extrapolated to those scenarios [34,131,157,158]. If the natural immunity against the SARS-CoV-2 virus diminishes over time, then the constructed mathematical model needs to be modified. Similarly, if the protection of the vaccine is shorter than 10 months, the model presented here needs to be modified in order to include the transition from *V*_2_(*t*) to the susceptible subpopulation *S*(*t*) or to a similar one with mild protection. Thus, overall new and more complex mathematical models are necessary to consider the appearance of new SARS-CoV-2 variants and waning immunity [21,30].

## 5. Conclusions

The design of optimal health policies and, therefore, of vaccination programs, is of paramount importance in order to reduce the burden of the COVID-19 pandemic. In this work, we developed a mathematical model of COVID-19 transmission to study the effect of different vaccination programs varying the time between the first and second doses. In addition, a variety of scenarios has been considered varying the efficacy of the first dose vaccine and the vaccination rate. Thus, we are able to gain insight regarding the optimal strategy for vaccination campaigns. We found that for vaccines with a high efficacy after the first dose, the optimal strategy is to delay the inoculation of the second dose. On the other hand, for a low first dose efficacy, it is better to use the standard vaccination regimen of 4 weeks between doses. Thus, under the delayed second dose option, the focus of the vaccination campaign for generating certain immunity in a great number of people as fast as possible instead of having an almost perfect immunity in a smaller percentage of people first has been shown by our results to be preferable. Thus, in this light, the UK implemented a better vaccination campaign than the USA. The results presented here provide guidelines for other countries where vaccination campaigns are just starting.

The results presented here are based on measuring certain health outcomes such as cumulative mortality, cumulative infected, and the cumulative number of hospitalizations. All these outcomes are related due to the intrinsic characteristic of the COVID-19 disease. The results presented here have the potential to improve the design of vaccination campaigns under constraints in the vaccine supply. Thus, health authorities can maximize population health benefits. The results obtained here suggest that a good vaccination policy would be to vaccinate more people with one dose as soon as possible in order to increase the protection provided by the vaccine. Based on the current high vaccine efficacies for the first dose, the proposed strategy seems logical, as the results have shown. However, as some researchers have mentioned, this could be risky, and further studies are necessary since the initial clinical trials used the standard vaccination regimen of 4 weeks between doses [15].

Modeling studies have inherent limitations, but despite the limitations and assumptions of the constructed mathematical model, the methodology presented here can be extrapolated to different countries with different conditions regarding social behavior and vaccine availability. All the results presented here are subject to the assumptions made for the mathematical model and parameter values. If in the near future new scientific knowledge regarding SARS-CoV-2 arises, then the results will need to be reevaluated. One important question that might be answered during the year 2021 is the one related to the durability of the efficacy and variability over the time of the vaccine against SARS-CoV-2. The answer to that question probably would require modifying the models that have been used to study a variety of vaccination campaigns. It is important to remark that models cannot forecast future non-pharmaceutical interventions nor future social behavior of the general population. However, we have considered different scenarios that help in this direction.

Finally, the results obtained in this work encourage health authorities to focus on deploying a vaccination campaign with a delayed second dose in order to provide certain protection to a larger population. Age groups under higher risk also should be prioritized using a similar approach to the delayed second dose, as a previous study suggested [22]. The results of this study agree with previous results regarding the benefits of a delayed vaccination regimen, despite the use of different modeling approaches and different starting assumptions [21-23]. Thus, these conclusions provide robustness and reliability to the strategy of delaying the second dose under plausible scenarios.

## Figures and Tables

**Table 1.** Initial conditions assumed for the different subpopulations.

Parameter	Symbol	Value
Latent	*E*(0)	997,600
Presymptomatic	*P*(0)	791,200
Infected (symptomatic)	*I*(0)	1,204,000
Asymptomatic	*A*(0)	1,204,000
Hospitalized	*H*(0)	71,552
Recovered	*R*(0)	16,462,937
Total population	*N*(0)	330,705,643

**Table 2.** Mean values of parameters used to perform numerical simulations of the different scenarios.

Parameter	Symbol	Value
Latent period	1/*α*	2.9 days [1,118,119]
Presymptomatic period	*1/p*	2.3 days [1,118,119]
Infectious period	1/*γ*	7 days [118]
Hospitalization rate	*h*	0.1/7 days^−1^ [4,118,120]
Hospitalization period	*ρ*	0.9/10.4 days^−1^ [4,118,120]
Death rate (hospitalized)	*δ*	0.1/10.4 days^−1^ [17,121]
Probability of being asymptomatic	*a*	0.5 [1,111]
Efficacy of the vaccines	*ε*_i_	Varied
